# Methicillin-Resistant *Staphylococcus epidermidis* Lineages in the Nasal and Skin Microbiota of Patients Planned for Arthroplasty Surgery

**DOI:** 10.3390/microorganisms9020265

**Published:** 2021-01-28

**Authors:** Emeli Månsson, Staffan Tevell, Åsa Nilsdotter-Augustinsson, Thor Bech Johannesen, Martin Sundqvist, Marc Stegger, Bo Söderquist

**Affiliations:** 1 School of Medical Sciencies, Faculty of Medicine and Health, Örebro University, SE-701 82 Örebro, Sweden; staffan.tevell@regionvarmland.se (S.T.); mtg@ssi.dk (M.S.); bo.soderquist@regionorebrolan.se (B.S.); 2 Centre for Clinical Research, Region Västmanland—Uppsala University, Hospital of Västmanland, Västerås, SE-721 89 Västerås, Sweden; 3 Department of Infectious Diseases, Karlstad Hospital and Centre for Clinical Research and Education, County Council of Värmland, SE-651 82 Karlstad, Sweden; 4 Department of Infectious Diseases, and Department of Clinical and Experimental Medicine, Linköping University, SE-60182 Norrköping, Sweden; asa.nilsdotter-augustinsson@liu.se; 5 Department of Bacteria, Parasites and Fungi, Statens Serum Institut, 2300 Copenhagen, Denmark; thej@ssi.dk; 6 Department of Laboratory Medicine, Clinical Microbiology, Faculty of Medicine and Health, Örebro University, SE-701 82 Örebro, Sweden; martin.sundqvist@regionorebrolan.se

**Keywords:** *Staphylococcus epidermidis*, microbiota, multidrug resistance, genome sequencing, phylogenetic analyses, arthroplasty surgery

## Abstract

*Staphylococcus epidermidis*, ubiquitous in the human nasal and skin microbiota, is a common causative microorganism in prosthetic joint infections (PJIs). A high proportion of PJI isolates have been shown to harbor genetic traits associated with resistance to/tolerance of agents used for antimicrobial prophylaxis in joint arthroplasties. These traits were found within multidrug-resistant *S. epidermidis* (MDRSE) lineages of multiple genetic backgrounds. In this study, the aim was to study whether MDRSE lineages previously associated with PJIs are present in the nasal and skin microbiota of patients planned for arthroplasty surgery but before hospitalization. We cultured samples from nares, inguinal creases, and skin over the hip or knee (dependent on the planned procedure) taken two weeks (median) prior to admittance to the hospital for total joint arthroplasty from 66 patients on agar plates selecting for methicillin resistance. *S. epidermidis* colonies were identified and tested for the presence of *mecA*. Methicillin-resistant *S. epidermidis* (MRSE) were characterized by Illumina-based whole-genome sequencing. Using this method, we found that 30/66 (45%) of patients were colonized with MRSE at 1–3 body sites. A subset of patients, 10/66 (15%), were colonized with MDRSE lineages associated with PJIs. The *qacA* gene was identified in MRSE isolates from 19/30 (63%) of MRSE colonized patients, whereas genes associated with aminoglycoside resistance were less common, found in 11/30 (37%). We found that MDRSE lineages previously associated with PJIs were present in a subset of patients’ pre-admission microbiota, plausibly in low relative abundance, and may be selected for by the current prophylaxis regimen comprising whole-body cleansing with chlorhexidine-gluconate containing soap. To further lower the rate of *S. epidermidis* PJIs, the current prophylaxis may need to be modified, but it is important for possible perioperative MDRSE transmission events and specific risk factors for MDRSE PJIs to be investigated before reevaluating antimicrobial prophylaxis.

## 1. Introduction

*Staphylococcus epidermidis* is ubiquitous in the human microbiota of the skin and mucosal membranes. *S. epidermidis* is in several ways beneficial to the human host, involved in the regulation of wound healing and defense against virulent pathogens such as *Staphylococcus aureus* [1], and is an important causative microorganism in healthcare-associated infections (HAI) [2], such as prosthetic joint infections (PJIs). PJIs are classified as acute hematogenous, early postoperative, or chronic [3] and are associated with significant morbidity [4] and mortality [5,6]. The international incidence of PJIs is estimated at 1% for total hip arthroplasties (THAs) and 1–2% for total knee arthroplasties (TKAs) [7]. Due to the rising number of arthroplasty surgeries performed annually, the number of patients with PJIs is increasing [8,9,10,11]. In early postoperative and chronic PJIs, coagulase-negative staphylococci (CoNS) are the most common causative microorganisms, reported in 25–29% of infections [12,13,14,15], and among CoNS, *S. epidermidis* is the most frequently reported species (61–85%) [16,17,18]. 

The pathogenesis of *S. epidermidis* PJIs is not fully understood, but bacteria may be introduced either intraoperatively during surgery or in the immediate postoperative period before the wound is completely healed [14]. Preventive measures to reduce the rate of PJIs include the optimization of modifiable host risk factors [19], the operating room environment [20], and systemic antimicrobial prophylaxis [21]. In Sweden, the national guideline for prevention of infections in prosthetic joint surgery (PRISS, https://lof.se/patientsakerhet/vara-projekt/priss/rekommendationer) recommends systemic antimicrobial prophylaxis with cloxacillin and surgery performed in operating theatres with ultra-clean air (<5 colony forming units/m^3^). In addition, preoperative skin cleansing with chlorhexidine-gluconate (CHG)-containing soap should be performed twice before surgery. Aminoglycoside-loaded bone cement of both components is also used in 60% of all primary THAs in Sweden [data from the Swedish Hip Arthroplasty Register (SHAR), www.shpr.se].

Previous molecular epidemiology studies have demonstrated that multidrug-resistant (MDR) *S. epidermidis* (MDRSE) lineages, such as sequence type (ST) 2, ST5, ST59, and ST215, are predominant in PJIs [22,23,24,25]. This was verified in a recent comparative genomics study in which our group, using genome-wide association methods on approximately 300 sequenced isolates, demonstrated that *S. epidermidis* from PJIs differed from isolates retrieved from nasal mucosa by harboring genetic traits associated with resistance to/tolerance of compounds used for infection prevention in prosthetic joint surgery, such as betalactams, aminoglycosides, and chlorhexidine [26]. This is worrisome, as methicillin-resistant *S. epidermidis* (MRSE) and MDRSE are associated with worse treatment outcomes in PJIs than susceptible *S. epidermidis* [27,28].

In contrast to the predominance of a limited number of MDRSE lineages found in PJIs, metagenomic sequencing has demonstrated considerable strain heterogeneity of *S. epidermidis* in the human microbiome [29]. Culture-based studies have demonstrated low rates (4–25%) of methicillin-resistance in CoNS from patients sampled prior to or on admission to orthopedic wards [30,31,32,33]. The majority of CoNS isolates in these studies were presumably *S. epidermidis*, but identification at the species level was generally not performed. Selective media were only used in two of these studies [31,33], and it is possible that colonization with MRSE (and MDRSE) before admission (in low relative abundance) is more common than previously thought. 

To further improve the prevention of *S. epidermidis* PJIs, it is important to investigate whether PJI-associated MDRSE lineages are present in the microbiota of patients scheduled for prosthetic joint surgery before hospital admission or whether these lineages are acquired during hospitalization. The hypothesis in this study was that MDRSE associated with PJIs is present in the pre-admission microbiota of arthroplasty surgery patients but in low relative abundance and thus is largely undetected by nonselective culturing methods. To test this, we sub-cultured samples from the nares, inguinal creases, and skin over the hip/knee on selective agar plates to optimize the retrieval of MRSE isolates, which were further characterized by genomic analyses. The aim was to study whether MDRSE lineages associated with PJIs are present in the nasal and skin microbiota of patients scheduled for prosthetic joint surgery before hospital admission.

## 2. Materials and Methods

One hundred patients scheduled for prosthetic hip- or knee joint replacement surgery were sampled from the nares, inguinal crease, and skin over the hip/knee (depending on the joint to be replaced) two weeks (median) before admission to a hospital using flocked swabs (ESwab, Copan Italia Spa, Brescia, Italy). Sampling was performed at three orthopedic outpatient clinics in central Sweden: A) Karlstad (regional hospital, *n* = 40; Jan 2013 to Jan 2014), B; Västerås (regional hospital, *n* = 37; Dec 2012 to May 2014), and C) Linköping (university hospital, *n* = 23; Mar 2013 to Jan 2015) (Figure S1), following informed consent. The number of primary THAs and TKAs performed annually at these hospitals was A, 457 (2013); B, 732 (2013); and C, 67 (2014) (data from SHAR and the Swedish Knee Arthroplasty Register). The study protocol was approved by the Regional Ethical Review Board of Uppsala, Sweden (project identification code 2012/092, 18/04/2012). 

### 2.1. Culture, DNA Extraction, and Illumina Sequencing

All samples were sent to the Department of Laboratory Medicine, Clinical Microbiology, Örebro University Hospital and subsequently plated on blood agar [Columbia II Agar 3.9% *w/v* (Oxoid, Basingstoke, Hampshire, UK)] supplemented with 6% defibrinated horse blood (SVA, Uppsala, Sweden)). All growths on the plates were harvested and stored frozen (−80 °C) in a preservation medium (trypticase soy broth, BD Diagnostic Systems, Sparks, MD, USA), supplemented with 0.3% *w/v* yeast extract (BD Diagnostic Systems) and 29% horse serum (SVA, Uppsala, Sweden). 

**Figure 1:**
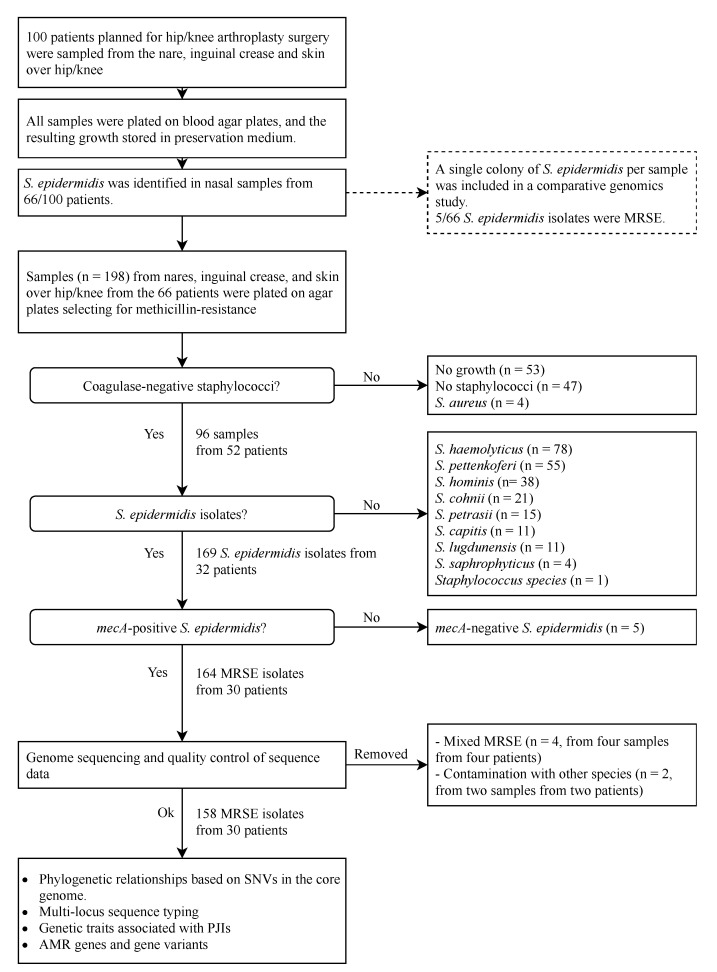
Origin of the isolates sequenced within this study and overview of analyses performed: one-hundred patients planned for arthroplasty surgery were sampled from their nares, inguinal creases, and skin over their hip/knee joints. *S. epidermidis* was retrieved from nasal samples plated on Müller–Hinton (MH) agar plates from 66 patients. A single colony of S. epidermidis was randomly chosen from the nasal sample of these 66 patients. Methicillin-resistant *S. epidermidis* (MRSE) was identified in samples from five out of the 66 patients. To investigate to what extent this underestimated the true colonization rate with MRSE, we plated samples from nares, inguinal creases, and skin over the hip/knee joints for these 66 patients on agar plates selecting for methicillin resistance. Coagulase-negative staphylococci were identified by colony morphology and species-determined using MALDI-TOF. The isolates identified as *S. epidermidis* were analyzed for the presence of *mecA*, and *mecA*-positive *S. epidermidis* isolates were further characterized by Illumina sequencing to determine the phylogenetic relationships, multilocus sequence types, genes associated with prosthetic joint infections (PJIs), and antimicrobial resistance (AMR) genes and gene variants.

In the present study, we included samples from the nasal mucosa, inguinal crease, and skin over either the hip or knee from 66 patients in whom *S. epidermidis* was identified in nasal samples after culturing on nonselective agar plates (Figure 1). From the frozen bacterial suspensions of the original subculture, 10 µL was cultured on selective media (Mueller-Hinton II agar 3.8% *w/v* (BD Diagnostic Systems) supplemented with 5 mg/L cefoxitin (Sigma Aldrich, Darmstadt, Germany) and 10 µL on a chromogenic medium primarily used for detecting methicillin-resistant *S. aureus* (MRSA) (CHROMID MRSA, bioMérieux, Marcy-l’Étoile, France). After 48 h of incubation in an aerobic atmosphere at 36 °C, five colonies (when available) with macroscopic colony morphology consistent with staphylococci were randomly chosen and identified to the species level using MALDI-TOF MS (Microflex LT, Bruker Daltonik, Bremen, Germany) with Biotyper 3.1 DB7311, DB7854, and DB8468 (Bruker Daltonik). The isolates identified as *S. epidermidis* were analyzed for the presence of *mecA* using PCR [34] or, in selected cases, loop-mediated isothermal amplification (LAMP, Genie II, Amplex Diagnostics GmbH, München, Germany and easyplex MRSAplus, Amplex). 

The DNA was purified using the Roche MagNA Pure 96 (F. Hoffman-La Roche Ltd., Basel, Switzerland) system after incubation overnight in 36 °C on blood agar plates (SSI Diagnostica, Denmark). The extracted DNA was quantified using the Qubit fluorometer (Invitrogen, Waltham, MA, USA), followed by library preparation using the Nextera XT DNA Library Prep Kit (Illumina Inc., San Diego, CA, USA), according to the manufacturer’s protocol. Sequencing was performed on a NextSeq 550 platform (Illumina Inc., San Diego, CA, USA) to obtain paired-end 151 bp reads. The generated sequencing data were subjected to quality control using bifrost (https://github.com/ssi-dk/bifrost) to ensure adequate sequencing depth of all isolates. Intraspecies contamination was checked using NASP v1.0.0 [35]. All genome sequences were archived at the European Nucleotide Archive under project ID PRJEB34788 with accession numbers ERR3585469–ERR3585625. 

### 2.2. Phylogenetic Analysis

**Figure 2:**
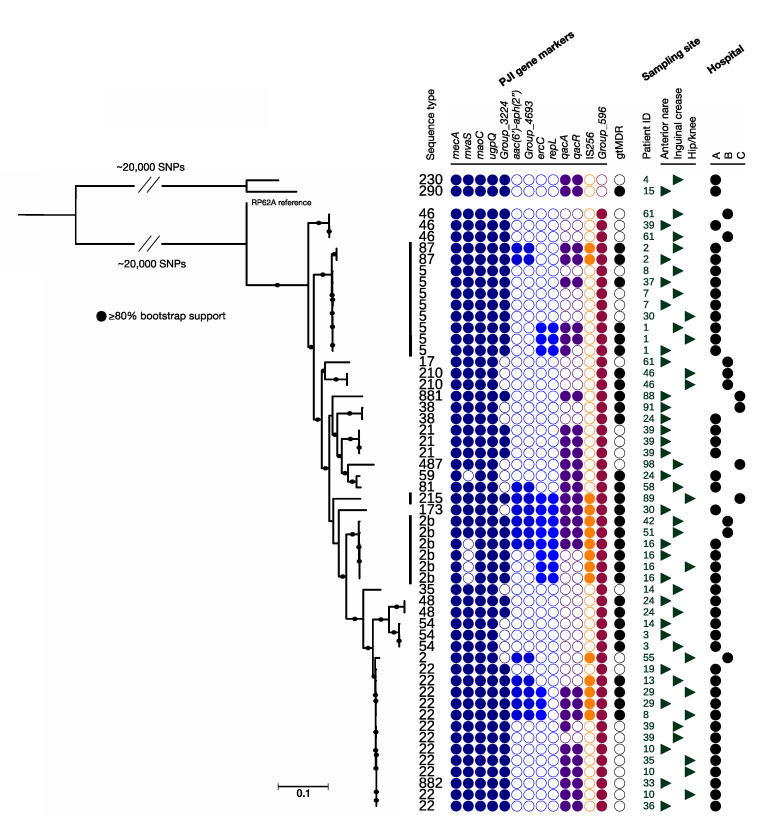
Phylogenetic relatedness of 55 *mecA*-positive *S. epidermidis* isolates retrieved from sampling of 66 patients prior to hospitalization for prosthetic joint surgery: the lineages previously associated with PJIs in Sweden (ST5, ST215, and ST2b) are indicated with a solid bar. Columns from left to right: sequence type (ST), genes previously associated with PJI origin (genes associated with SCC*mec* [dark blue], aminoglycoside resistance [medium blue], macrolide-lincosamide resistance [light blue], chlorhexidine tolerance [purple], IS*256* [orange], and genes coding for proteins of unknown function [dark red]), genotypic multidrug-resistant (black), patient running number, sample origin (body area), and hospital. The mid-point rooted phylogeny was based on 66,152 SNPs, and the scale bar indicates substitutions per site.

Sequencing data were aligned to the *S. epidermidis* RP62a reference chromosome (GenBank accession number NC_002976.3) using NASP v1.0.0 [35] with BWA-MEM v0.7.12 [36] and subsequent single nucleotide polymorphism (SNP) calling using GATK [37]. If a variant was present in <90% of the base calls per site per individual isolate or a minimum coverage of 10 was not met, the position was excluded across the collection to retain only high-quality variant callings. Phylogenies were obtained with RAxML v8.2.1 [38] using the GTRCAT model and 100 replicates for bootstrapping. The sequence data from four *S. epidermidis* PJI isolates representing the major PJI-associated lineages ST2a, ST2b, ST5, and ST215 were included in the phylogenetic analysis comprising all isolates. Within-sample isolates with ≤3 single nucleotide variant (SNV) distances were regarded as representative multiple isolates of the same strain [39,40]. A single isolate per strain and sample (the sequence with the highest base-pair coverage vs. the reference) was retained, and a phylogenetic tree depicting diversity of MRSE strains retrieved from the nares, inguinal crease, and skin area over the hip or knee before hospitalization was inferred (Figure 2).

### 2.3. Identification of Genes and Gene Variants Associated with Antimicrobial Resistance

After assembly using SPAdes [41], the genes and their location in the assemblies were identified using Prokka [42]. ABRicate (https://github.com/tseemann/abricate) was used to search the assembled genomes for genes associated with betalactam (*mecA*), aminoglycoside (*aac(6′)-aph(2′′*) and *aadD*), fusidic acid (*fusB* and *fusC*), and macrolide-lincosamide (*erm*(A), *erm*(C), *lnu*(A), *mph*(C), *msr*(A), *vat*(B), *vga*(A), and *vga*(B)) resistance present in the ResFinder database [43] accessed on 17 June 2019. The gene presences were determined based on a >80% hit length and >90% sequence identity. Previously identified gene variants associated with fusidic acid (*fusA*) [44], rifampicin (*rpoB*) [45,46], fluoroquinolone (*gyrA, grlA,* and *grlB*) [47,48], and trimethoprim/sulfamethoxazole (*dfrG* and F99Y variants of *folA*) [49] resistance were investigated using BLASTN searches in Biomatters Geneious Prime v2019.2.1 (Biomatters Ltd., Auckland, NewZealand) as previously described [26]. Isolates with the presence of genes and/or point mutations associated with resistance towards ≥3 of the following antimicrobial categories were defined as multidrug-resistant (MDR): methicillin, fusidic acid, macrolide-lincosamide, rifampicin, aminoglycosides, fluoroquinolones, and trimethoprim/sulfamethoxazole. 

### 2.4. Identification of STs

Allelic profiles and sequence types were assigned using *mlst* (https://github.com/tseemann/mlst) based on the PubMLST typing scheme for *S. epidermidis* (https://pubmlst.org/sepidermidis/) [50]. ST2 was divided into ST2a and ST2b lineages based on previous findings [26]. 

### 2.5. Identification of Genetic Traits in S. epidermidis Associated with PJIs

Genes in *S. epidermidis* previously associated with PJIs [26] were determined using Mykrobe [51] on raw sequencing data as previously described [26]. Genes were identified using 90% sequence length and a minimum coverage of five as the cutoffs. 

### 2.6. Statistics

The Chi-square test and Student’s *t*-test were used to test the differences in the MRSE colonization rates and in mean age between the patients recruited between the three hospitals. Fisher’s exact test was used to test the differences in genotypic antimicrobial resistance in MRSE from different body areas. A *p*-value (two-sided) < 0.05 was considered statistically significant. All statistical analysis was performed using IBM SPSS Statistics v25 except for the normal approximation confidence interval for the percentage of patients colonized with MDRSE, which was calculated using www.openepi.com v3.01.

## 3. Results

A total of 198 samples from 66 patients (33 women and 33 men) sampled at a median of 15 [interquartile range (IQR) 10.0–41.0] days before arthroplasty surgery (46 THAs and 20 TKAs) were included in the study. The mean age, which was 67 years (SD 10.4) for the entire study population, differed between hospitals: patients recruited from regional hospital A were older [mean age 70.6 (SD 10.8)] than patients recruited from university hospital C [mean age 59.4 (SD 7.7)] (*p* = 0.0004). 

In total, 403 CoNS, originating from 96 samples from 52 patients, were identified on any of the methicillin-resistance selective agar plates. No growth was found on any of the agars for 53 (27%) samples [nares, *n* = 20 (30%); inguinal crease, *n* = 8 (12%); and hip/knee, *n* = 25 (38%)], and no colonies with the macroscopic appearance of staphylococci were found in the 47 samples (Figure 1). The most frequently identified CoNS species was *S. epidermidis* (*n* = 169, 33 patients), followed by *S. haemolyticus* (*n* = 78, 18 patients), *S. pettenkoferi* (*n* = 55, 10 patients), *S. hominis* (*n* = 31, six patients), *S. cohnii* (*n* = 21, four patients), *S. petrasii* (*n* = 15, five patients), *S. capitis* (*n* = 11, four patients), and *S. lugdunensis* (*n* = 11, four patients) (Figure 1). One isolate, identified at the genus level as a *Staphylococcus* species by DB7311 and DB7854, was not available for identification with DB8468 as it had succumbed in the freezer. *S. aureus* was retrieved from two samples from two patients; none of these isolates was *mecA/C* positive. 

Out of 169 *S. epidermidis* isolates, 164 isolates originating from 44 samples from 30 patients (30/66, 45% of patients, 95% C.I. 33–57%) were verified to be *mecA*-positive and were further characterized by genome sequencing. The colonization rates with MRSE varied between hospitals: A, 20/29 (69%); B, 6/19 (32%); and C, 4/18 (22%) (*p* = 0.003, df = 2). MRSE was retrieved from a single body site in 17 of the 30 patients colonized with MRSE: seven patients in the nasal samples, six patients in the inguinal crease samples, and four patients from the skin over the joint that was to be replaced (Table 1). In samples from 11 patients, MRSE was retrieved from the nasal mucosa and either the inguinal crease (*n* = 7) or site of planned surgery (*n* = 4); in one MRSE-colonized patient, MRSE was retrieved from the inguinal crease and the site of planned surgery but not from the nasal sample; and in one patient, MRSE was retrieved in all three samples (Table 1).

Following quality control of the sequence data, six isolates were excluded, four due to signs of intraspecies contamination based on the failed proportion filter using NASP and two due to contamination with other species using Kraken as implemented in bifrost. In total, 157 MRSEs were included in an initial phylogenetic analysis. Based on a conserved core genome of 1.8 Mbp (69.7%), 52 MRSE strains (isolates from the same patient separated by 0–3 SNVs) were identified in the 44 samples [1–4 MRSE strain(s) per sample; Table 1]. As presented above, 13 patients were colonized with MRSE at multiple sites (Table 1). A detailed phylogenetic analysis demonstrated that the same MRSE strain (separated by 0–3 SNVs) was retrieved from two body sites in three patients and that similar strains (here, defined as separated by ≤40 SNVs) were found at multiple sites in five patients. Six patients were colonized with MRSE that differed more than 40 SNVs between the sampled sites (range 3917–8466 SNVs); see Table 1. Twenty-three STs were identified among the 52 MRSE strains, including two novel STs: ST881 and ST882. 

To visualize the overall relatedness of MRSE strains retrieved from pre-admission sampling, a second phylogeny including only a single isolate per MRSE strain per sample (*n* = 55; identical strains were found in two samples for three patients) was constructed (Figure 2).

The MRSE belonging to PJI-associated lineages were identified in samples from ten patients, ST2b lineage, *n* = 3; ST5 lineage, *n* = 6; and ST215 lineage, *n* = 1, while no isolate belonging to the ST2a lineage was found. Nine patients were colonized with MRSE isolates belonging to the ST22 lineage, and one patient was colonized with the ST2 isolate that was closely related to the ST22 lineage (previously referred to as ST2c [26]).

The genes previously associated with PJI in *S. epidermidis* [26] are presented in Figure 2. The *aac(6′)-aph(2′′)* gene (associated with aminoglycoside resistance) was present in MRSE isolates from 11/30 MRSE-colonized patients (37%), *ermC* (associated with macrolide-lincosamide resistance) was present in eight (27%) patients, and *qacA* (associated with chlorhexidine tolerance) was present in 19 (63%) patients. The transposable element IS*256* was present in MRSE isolates from 10 patients (33%). The *group_596* gene was found in 50 out of the 52 MRSE strains (28/30 MRSE colonized patients).

The genes and gene variants associated with resistance towards fluoroquinolones, fusidic acid, macrolides-lincosamides, rifampicin, and trimethoprim-sulfamethoxazole were determined in the MRSE isolates. The isolates within a strain displayed the same pattern of AMR gene absence/presence in all but two cases. The frequency of genes and/or gene variants associated with antimicrobial resistance is presented per MRSE positive sample by antimicrobial category in Table 2. No mutations associated with rifampicin-resistance was found in any of the MRSE isolates. No statistically significant difference in resistance towards different antimicrobial agents was found between samples from the nares, inguinal crease, and skin over the hip/knee. Overall, MDRSE was retrieved from 19 patients (29%).

## 4. Discussion

In this study, the aim was to investigate whether MDRSE lineages associated with PJIs are present in the nasal and skin microbiota of patients scheduled for prosthetic joint surgery before hospital admission. HAI with MDRSE is a growing concern, but unlike MRSA [52], there are no guidelines recommending the surveillance of MRSE and/or MDRSE [53]. The possible transmission events of MDRSE still remain unclear, and epidemiological studies of the prevalence of MDRSE in patients at risk of *S. epidermidis* infections has been called for [54]. To our knowledge, this is the first study using whole-genome sequencing to characterize MRSE isolates in the microbiota of patients scheduled for prosthetic joint surgery. We show that a subset of patients (10/66) were colonized with MDRSE lineages associated with PJIs [22,26] and that a number of patients were colonized with MDRSE lineage ST22 (a single locus variant of ST2), represented in publicly available international *S. epidermidis* sequences from PJIs [26]. All ST22-colonized patients were recruited from hospital A, where no data on the molecular epidemiology of *S. epidermidis* in PJIs was available. Overall, MRSEs were retrieved from almost half of patients in this study.

The majority of MRSE-colonized patients (19/30) were colonized with MRSE in the nares, but just over one third (11/30, including 4/10 patients colonized with lineages that predominate in PJIs) were not, suggesting that nasal screening alone is insufficient to detect individuals colonized with MRSE before surgery. Furthermore, we found that colonization with MRSE is likely to be vastly underestimated when a culture-based method using nonselective media is used. In the present study, we used samples from patients from whom *S. epidermidis* had already been identified from nasal samples after culturing on nonselective media. MRSE was only identified in 8% (5/66) of nasal samples using nonselective media, whereas by using selective media, we detected MRSE in nasal samples from an additional 14 patients (in total 29%, 19/66) and from an additional 11 patients from other body areas, summing up to a MRSE colonization rate of 45% (30/66 patients). This indicates that the vast majority of patients colonized with MRSE are missed when a single *S. epidermidis* colony is randomly chosen from a culture of a nasal swab plated on nonselective media.

The proportion of patients colonized with MRSE varied between the three hospitals. This difference could plausibly be explained by differences in prior hospitalization, as colonization rates of methicillin-resistant CoNS has been found to increase from 4–25% to 25–81% during hospitalization for orthopedic surgery [30,31,32], and almost twice as high carriage rates of methicillin-resistant CoNS have been found in patients undergoing revision THA (46%) compared with primary THA (24%) [55]. In this study, we had no access to data regarding prior admissions, but patients recruited from hospital A (with the highest MRSE colonization rate) were significantly older than those recruited from hospital C (with the lowest MRSE colonization rate), supporting the possibility that prior hospitalization rates may have differed between hospitals.

Preoperative whole-body cleansing with CHG-containing soap at home is recommended in national guidelines for prosthetic joint surgery in Sweden and is supported by the International Consensus Meeting on Musculoskeletal Infections despite a lack of robust evidence [56]. The increased tolerance of *S. epidermidis* to CHG is associated with the gene *qacA* that encodes an efflux pump [57], and *qacA* was one of the genetic traits associated with *S. epidermidis* isolated from PJIs in our previous study [26]. Here, where samples were obtained before whole-body cleansing with CHG-containing soap, almost one third of patients (19/66, 29%) were colonized with MRSE strains harboring *qacA*, possibly in low relative abundance. As *qacA* was found to be rare in methicillin-susceptible colonizing isolates [26], it can be speculated that the relative proportion of MDRSE in the microbiota after preoperative whole-body cleansing with CHG-containing soap may increase. This could subsequently increase the risk of perioperative contamination of the wound and subsequently the prosthetic joint with *S. epidermidis* resistant to standard systemic and locally administered antimicrobial prophylaxis. However, this remains to be explored, and most patients were not colonized with *qacA*-positive MRSE before hospital admission.

The prevalence of the remaining genes previously associated with *S. epidermidis* from PJIs varied. IS*256* and *ermC* were rare in general and almost exclusively found in MDR isolates, whereas *group_596,* encoding a hypothetical protein, was frequently present. The *aac(6′)-aph(2′’)* gene encoding aminoglycoside resistance was rare among MRSE isolates other than those belonging to the ST2b, ST5, ST22, and ST215 lineages. This suggests that gentamicin in bone cement is effective for most MRSE strains that patients harbor before prosthetic joint surgery and, thus, can be an important supplement to systemically administered antimicrobial prophylaxis. The reported adjusted risk of revision is lower for cemented than uncemented THA in Sweden [58], but in a previous large registry-based study from Norway, the use of bone cement was associated with increased infections rates, as the rate of revision due to infection was increased for cemented procedures in which no antibiotics was admixed into the bone cement compared to uncemented procedures. However, an equal risk of revision for infection was found for uncemented and cemented hip arthroplasties with antibiotic-loaded cement [59].

The present study has several limitations. We excluded patients from whom *S. epidermidis* was not retrieved in nasal samples cultured on unselective media. However, the colonization rate of MRSE may have been underestimated from (i) MRSE outgrown by other species and/or methicillin-sensitive *Staphylococcus epidermidis* in the first culture on standard media, (ii) the use of a relatively small sample volume for selective culturing, (iii) the choice of cefoxitin concentration in the supplemented plates, and (iv) restricting sampling to five colonies with CoNS appearance per plate. Metagenomic sequencing of the samples could have been used to overcome these limitations related to culturing but would not have been able to give the same strain-level resolution and link mobile genetic elements to specific lineages. A targeted genomic approach would, however, be interesting and useful for further studies. Another limitation is that no data on previous hospital admissions or stays in long-term care facilities was available.

*S. epidermidis* has been called an “accidental pathogen” as its virulence determinants are also important for colonization, and *S. epidermidis* infections have been regarded as resulting from haphazard contamination by isolates from the microbiota [60]. In this study, we show that a significant proportion of patients scheduled for prosthetic joint surgery was colonized with heterogeneous MRSE strains. Most of the MRSE strains lacked genes encoding resistance to gentamicin and/or tolerance of CHG, compounds used for the prevention of PJIs in Sweden, but a subset of patients were colonized with PJI-associated MDRSE lineages not covered by current prophylaxis guidelines. To lower the rate of PJIs, broader antimicrobial prophylaxis with a dual regimen of a betalactam and a glycopeptide to cover methicillin-resistant isolates has been suggested [61]. However, as prosthetic joint surgery is a high-volume surgery, broader antimicrobial prophylaxis would risk further selection of resistance traits in MDRSE circulating within and between hospitals.

If MDRSE-colonized patients can be demonstrated to have a higher risk of *S. epidermidis* PJIs, screening for MDRSE before surgery could be of interest to limit the use of broader antimicrobial prophylaxis to these patients only [33]. However, we also acknowledge that hospital-acquired re-colonization with MDRSE after preoperative whole-body cleansing with CHG-containing soap can be important and that possible transmission of MDRSE between patients, the hospital environment, and healthcare workers is an important issue for future research.

## 5. Conclusions

We found that MDRSE lineages previously associated with PJIs are present in a subset of patients’ pre-admission microbiota, plausibly in low relative abundance, and could be selected for by the current prophylaxis regimens. To further lower the rate of *S. epidermidis* PJIs, the current prophylaxis may need to be modified, but it is important for possible perioperative MDRSE transmission events as well as specific risk factors for MDRSE PJIs to be investigated before reevaluation of antimicrobial prophylaxis and/or screening and eradication of MDRSE/MRSE.

## Figures and Tables

**Table 1:** Sequence-types (STs) of methicillin-resistant *S. epidermidis* (MRSE) per patient and sample (nasal/inguinal crease/skin over hip/knee): number in bold indicates multidrug-resistant MRSE. Intra-patient single nucleotide variant (SNV) distances between samples (far right) highlight that MRSE with identical STs differ by 1–40 SNVs between two samples from the same patient.

				STs of MRSE Strains per Sample			
Patient	Age	Sex	Hospital	Nasal Mucosa	Inguinal Crease	Hip/Knee	SNV Distance between Samples ^1^
1	84	M	A	**5**	**5**	**5**	6–40
2	83	M	A	**87**	**87**	−	2
3	80	F	A	**54**	**54**	−	12
4	79	F	A	−	230	−	
7	76	F	A	5	5	−	1
8	75	M	A	−	5	**22**	3917
10	75	F	A	22	−	22, 22	13–20
13	73	F	A	−	**22**	−	
14	71	M	A	**54**	35	−	3917
15	71	F	A	**290**	−	−	
16	70	F	A	**2, 2, 2**	−	**2**	9–10
19	61	M	A	22	−	−	
24	54	F	A	**48, 38, 59**	**48**	−	3–8466
29	43	F	A	**22**	−	**22**	19
30	73	F	A	**173**	−	5	5842
33	78	F	A	882	−	−	
35	73	F	A	−	−	22	
36	60	F	A	22	−	−	
37	83	F	A	**5**	−	−	
39	74	F	A	21, 21, 21, 46	22, 22	−	5641–6381
42	77	M	B	−	**2**	−	
46	70	M	B	−	−	**210, 210**	
51	61	F	B	−	**2**	−	
55	60	M	B	−	−	2	
58	81	M	B	−	**81**	−	
61	57	F	B	17	46, 46	−	5411–5414
88	46	M	C	**881**	−	−	
89	64	M	C	−	−	**215**	
91	49	M	C	**38**	−	−	
98	53	F	C	−	487	−	

^1^ Range when MRSE was found in more than one sample.

**Table 2:** Co-resistance (based on the presence of gene(s)/gene variants) to other antimicrobial agents in methicillin-resistant *S. epidermidis* (MRSE) retrieved from sampling of patients planned for prosthetic joint surgery prior to hospital admission (data are presented as the number (percentage) of samples with resistant MRSE). n.s = not significant.

Antimicrobial Agent	Nasal Mucosa (*n* = 19)	Inguinal Crease (*n* = 15)	Hip/Knee (*n* = 10)	Total	
Aminoglycosides	6 (32%)	5 (33%)	5 (50%)	16 (36%)	n.s
Fluoroquinolones	7 (37%)	6 (40%)	5 (50%)	18 (41%)	n.s
Fusidic acid	6 (32%)	2 (13%)	1 (10%)	9 (20%)	n.s
MLS ^1^	12 (63%)	9 (60%)	7 (70%)	28 (63%)	n.s
Rifampicin	0	0	0	0	
TMP/SMX ^2^	9 (63%)	5 (33%)	4 (40%)	18 (41%)	n.s
Multidrug-resistant	12 (63%)	8 (53%)	6 (60%)	26 (59%)	n.s

^1^ macrolide-lincosamide-streptogramin; ^2^ trimethoprim-sulfamethoxazole.

## Data Availability

The data presented in this study are openly available in the European Nucleotide Archive under project ID PRJEB34788 with accession numbers ERR3585469–ERR3585625.

## Supplementary Materials

The following are available online at https://www.mdpi.com/2076-2607/9/2/265/s1, Figure S1: Map of central Sweden; patients were recruited from Karlstad (regional hospital), Västerås (regional hospital), and Linköping (university hospital).
