# Supplementary figures and images for: Methicillin-Resistant Staphylococcus epidermidis Lineages in the Nasal and Skin Microbiota of Patients Planned for Arthroplasty Surgery
Source: Microorganisms. 2021 Jan 28;9(2):265. doi: 10.3390/microorganisms9020265 (PMC7911009; doi:10.3390/microorganisms9020265)

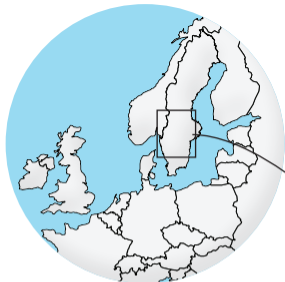

1000 km

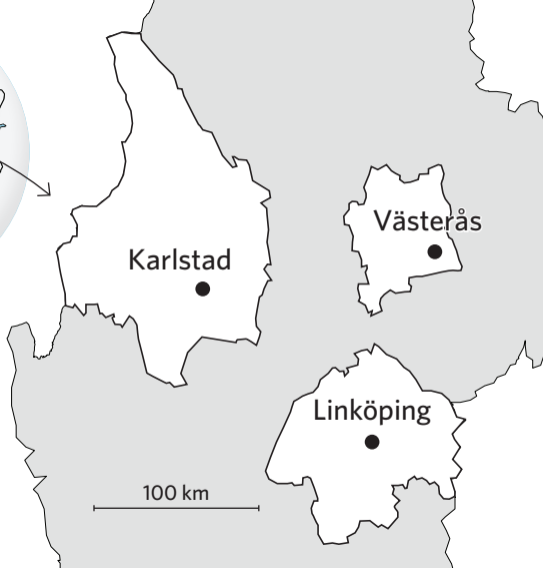

Karlstad

Västerås

Linköping

100 km

Supplement: Supplementary file 1 [file microorganisms-09-00265-s001.pdf]
